# Factors That Influence Sustained Release from Hot-Melt Extrudates

**DOI:** 10.3390/pharmaceutics15071996

**Published:** 2023-07-20

**Authors:** Yaser Mansuroglu, Jennifer Dressman

**Affiliations:** Fraunhofer Institute of Translational Medicine and Pharmacology, Theodor-Stern-Kai.7, 60596 Frankfurt am Main, Germany; yaser.mansuroglu@itmp.fraunhofer.de

**Keywords:** hot-melt extrusion, sustained release, dissolution, particle size, Eudragit polymer, flurbiprofen

## Abstract

Hot-melt extrusion is a well-established tool in the pharmaceutical industry, mostly implemented to increase the solubility of poorly soluble drugs. A less frequent application of this technique is to obtain formulations with extended release. This study investigated the influence of polymer choice, drug loading, milling and hydrodynamics on the release of a model drug, flurbiprofen, from sustained-release hot-melt extrudates with Eudragit polymers. The choice of polymer and degree of particle size reduction of the extrudate by milling were the two key influences on the release profile: the percentage release after 12 h varied from 6% (2 mm threads) to 84% (particle size <125 µm) for Eudragit RL extrudates vs. 4.5 to 62% for the corresponding Eudragit RS extrudates. By contrast, the release profile was largely independent of drug loading and robust to hydrodynamics in the dissolution vessel. Thus, hot-melt extrusion offers the ability to tailor the release of the API to the therapeutic indication through a combination of particle size and polymer choice while providing robustness over a wide range of hydrodynamic conditions.

## 1. Introduction

Hot-melt extrusion is a well-established tool in the pharmaceutical industry, mostly implemented to increase the solubility of poorly soluble drugs [1,2,3]. In this process, a mixture of the active pharmaceutical ingredient (API), a polymer (often based on acrylates, lactic acid, or cellulose derivatives) and a plasticizer, e.g., stearic or triethyl citric acid, is continuously fed into a single or twin-screw extruder and melted at a specific temperature. Simultaneously, the mixture is kneaded by the screws and thereby homogenized. Drug-loaded filaments are produced by propagating the mixture through a die with a defined diameter [1,2].

These filaments can be processed further to produce various shapes and sizes. For example, the filaments can be cut into equal-sized pieces while still soft and then spheronized to produce pellets. They can be milled to produce powders of defined particle size [2,3,4,5,6,7,8] or, in the most recent applications, used in the 3D printing of solid drug formulations via fused deposition modeling [9,10,11,12]. Depending on the choice of polymer, hot-melt extrudates can be used to improve the bioavailability of poorly soluble drugs, as biodegradable/non-biodegradable drug-loaded implants or, less often, as oral sustained-release dosage forms [2,3,4,5,6,7,8].

The aim of the current project was to understand how various formulation parameters influence the dissolution performance of sustained-release hot-melt extrudates using flurbiprofen as a model drug. Flurbiprofen, an NSAID (non-steroidal anti-inflammatory drug), is well established for use as a spray or lozenge to alleviate sore throat (e.g., Dobendan Direkt©) and in oral dosage forms (e.g., Froben© tablets, capsules) for the therapy of rheumatic diseases [13,14,15,16]. In the latter case, sustained release of flurbiprofen would be advantageous in attaining a constant therapeutic effect and reducing the frequency of administration since this API has a short half-life of around 5 h [17].

Poorly soluble Eudragit polymers and those with pH-dependent solubility are often used in oral pharmaceutical products as a coating material to modify drug release from a dosage form. Those with pH-dependent solubility are also widely used in hot-melt extrusion (HME) and 3D printing [1,2,3,4,5,9,10,11,12]. In this research, Eudragit RL and RS were used to obtain sustained-release solid oral dosage forms by HME processing. These two Eudragit polymers are both non-erodible, cationic polymers belonging to the polymethacrylate family. Both are insoluble in water and release the active pharmaceutical ingredient (API) via diffusion in a pH-independent manner [2,18]. Both polymers, either alone or in mixtures at different ratios, have been widely implemented for the formulation of sustained-release dosage forms, mostly in film coatings [2,18,19,20,21,22,23,24,25,26,27,28,29]. While their molecular structure is nearly the same, the number of ammonium groups and, therefore, the permeability of these two polymers differ [2,18].

In these studies, flurbiprofen/Eudragit extrudates were investigated to assess (a) the ability of the extrudates to retard API release, (b) the effect of particle size on the dissolution performance and (c) development aspects such as extrudability, maximum achievable drug load, homogeneity, and extent of (re)-crystallization. To date, no publications have appeared in the literature addressing all these points systematically for hot-melt extrudates with Eudragit RL and RS. The dissolution of an API from a hot-melt extrudate using Eudragit RL or RS should depend mainly on its diffusion through the polymer to the surface of the extrudate. As the surface area, and consequently, the particle size, affects the diffusion rate of a molecule, the relationship between the particle size of the extrudate and the release pattern of the API from the extrudate is of particular interest.

## 2. Materials and Methods

### 2.1. Materials

Flurbiprofen, 98% pure (as the racemate), was purchased from Alfa Aesar (Thermo Fisher GmbH, Kandel, Germany). Eudragit RL PO and RS PO were kindly donated by Evonik Industries AG. Talcum, magnesium stearate and stearic acid were obtained from VWR chemicals (Darmstadt, Germany). The commercial product Cebutid^®^ LP 200 mg (Batch number: 2002) was obtained from Almirall SAS (Paris, France). FaSSIF V1 powder was purchased from Biorelevant.com Ltd. (London, United Kingdom), and FaSSIF V1 was prepared using the standard operating procedures provided by Biorelevant.com Ltd. (Biorelevant.com). Acetonitrile and TFA (Trifluoroacetic acid) used for HPLC analysis were obtained commercially from VWR chemicals (Darmstadt, Germany) and Merck KGaA (Darmstadt, Germany), respectively.

### 2.2. Methods

#### 2.2.1. Hot-Melt Extrusion

In pilot studies, different mixtures of flurbiprofen and other components were tested to identify the optimal composition for the extrusion process. Extrusion-relevant properties of the drug and polymers are shown in Table 1. The various compositions used in the pilot studies are shown in Table 2.

The initial choices of drug and excipient levels for the pilot studies were inspired by preliminary, in-house testing. In the pilot studies, a series of hot-melt extrusions using the components shown in Table 2 was first performed using an outlet temperature of 97.5 °C and a screw speed of 50 rpm. At the same time, a 2.0 mm rod die was deployed to obtain a fine cylindrical extrudate thread. Next, these parameters were optimized to an outlet temperature of 90 °C and a screw speed of 10 rpm during the release of the extrudate to improve the handling of the released extrudate. The slower screw speed during the final extrusion step facilitated the production of more uniform, straight threads.

Magnesium stearate is often used as a lubricant/filler material in HME of various polymers [36] to improve the handling of extrudate threads. In our pilot studies, it had a slight plasticizing effect on the extrudates and thus facilitated the extrusion process. Talcum, which is also used as a lubricant in HME [36], improved the mixing of the components inside the extruder as well as the handling of the extrudate threads and led to more homogeneous extrudates. Nevertheless, the extrudates were still too brittle for further processing. For this reason, stearic acid was substituted for magnesium stearate at a level of 4% m/m [2,23,30,36], and the level of talcum was also increased to 4% m/m to optimize processing during and after extrusion.

Due to the poor appearance of the pilot extrudates at a 33% m/m drug load of flurbiprofen (see results Section 4.1), drug loads were subsequently limited to the range 20–35% m/m for both Eudragit RL and RS. The optimized compositions at several drug loads are shown in Table 3. In each case, they were made by weighing the components (total weight 12 g), mixing them for 5 min with a mortar and pestle and then extruding with a conical co- and counter-rotating twin-screw extruder (Thermo Fischer Scientific Haake Minilab II, Dreieich, Germany, 60 × 30 × 40 cm, L/D ratio: 25/1, barrel volume 7 cm^3^,). Extrusion settings were 120 °C as the barrel temperature and 80 rpm as the screw speed. Before release from the extruder, the extrudate was cycled in the extruder for a dwell time of 5 min. To check the reproducibility of the hot melt process, two batches of each formulation were prepared and subjected to dissolution testing after ensuring that they met the critical quality attributes (CQA) of the batch with respect to the appearance and homogeneity of the API content.

#### 2.2.2. Homogeneity of Flurbiprofen Content in the Extrudates

To check the homogeneity of the flurbiprofen content in the formulations, the extrudate thread was cut into three segments of equal length, representing the start, middle and end of the extrusion process. Samples from each segment (*n* = 3), with a weight of approx. 1 mg apiece, were transferred into 10 mL volumetric flasks, filled to volume with mobile phase and dissolved by ultrasonication for at least 30 min. After appropriate dilution with mobile phase (1:1), the samples were analyzed by HPLC. For the API content, the limit of acceptable variation was set at ±5%.

#### 2.2.3. Differential Scanning Calorimetry (DSC)

To check the degree of crystallinity, samples of the pure API, excipients, physical mixtures of Eudragit RL and RS formulations (both at a 25% m/m drug load) and all the extrudates were analyzed by DSC (DSC 6000, Perkin Elmer, Waltham, MA, USA). The samples, each with a weight of ~6 mg, were weighed into aluminum pans, which were sealed with a thin pre-pierced lid (Perkin Elmer, Waltham, MA, USA), and then heated to 200 °C at a rate of 5 °C/min. The sample was then cooled back to 25 °C at the same rate.

#### 2.2.4. X-ray Powder Diffraction Analysis (XRPD)

To determine the degree of crystallinity, samples of pure API, excipients and extrudates were analyzed by XRPD. Each sample was analyzed over 2–70 theta.

#### 2.2.5. Milling of the Extrudates into Various Particle Size Ranges

The extrudates containing 25% m/m flurbiprofen were subjected to milling, after which they were classed into various particle size ranges. Fraction 1 consisted of 2 cm pieces (*n* = 3) of the extrudate thread, which were cut with a sharp scalpel, weighed (average weight ~100 mg) and subjected to dissolution testing. For fractions 2–6, the extrudate was milled repeatedly with an IKA^®^ tube mill (IKA^®^-Werke GmbH & CO. KG, Staufen, Germany) at 5000 rpm for 30 s. Between each milling step, the extrudates were cooled at −20 °C for about 5 min to prevent them from becoming sticky during the milling process. After milling, the extrudate was separated into six different particle size fractions using pharmaceutical test sieves with diameters ranging between 125 µm and 2 mm. Fraction 7 was obtained by milling the extrudate at 25,000 rpm for 1 min and sieving through a 125 µm sieve. The particle size range of each fraction is shown in Table 4. Prior to dissolution testing, the fractions were stored at −20 °C.

#### 2.2.6. Dissolution Testing

The parameters for the dissolution test were based on the tests described in the *USP monograph Flurbiprofen Tablets* [37] and the *Ph. Eur. 2.9.3 Chapter Dissolution Test for solid dosage forms* [38]. Two batches of each final formulation were tested (*n* = 3 or more per batch with 200 mg extrudate tested per vessel), while the commercial product (Cebutid LP© 200 mg) was tested at *n* = 6. The tests were performed in a calibrated USP apparatus 2 (Pharmatest PTWS 120S, Pharmatest AG, Hainburg, Germany). Instead of 900 mL of phosphate buffer pH 7.2, as prescribed in the monograph, 500 mL of degassed and filtered FaSSIF V1 biorelevant media, maintained at a temperature of 37 ± 0.5 °C, was used as the dissolution medium, as per Klumpp et al. [39]. The paddle rotation speed was 50 rpm, except for experiments specifically designed to explore the effect of hydrodynamics on the release rate. Samples were withdrawn at 15, 30, 45, and 60 min and 2, 4, 6, 8, 10 and 12 h. The samples were filtered through 0.1 µm PP filters (VWR, Leuven, Belgium), diluted with mobile phase (1:1) and analyzed by HPLC.

#### 2.2.7. HPLC Analysis

Quantification of flurbiprofen in extrudates and dissolution samples was performed using an EliteChrom Hitachi HPLC system (VWR, Leuven, Belgium) equipped with a Lichrocart 250-4, 100 RP 18, 5 µm, 250 × 4 mm column (Merck KGaA Darmstadt, Germany). Further details of the HPLC method are shown in Table 5.

#### 2.2.8. Data Presentation and Statistics

Dissolution data are reported as the mean and standard deviation of % release at each timepoint for all batches studied. The results of the dissolution measurements were compared using analysis of variance (ANOVA). For the calculation of the *p*-values with ANOVA (Microsoft Excel for Microsoft 365, Version 2208, Microsoft, Redmond, WA, USA), the dissolution values of the tested samples at a sampling time of 720 min were used. Dissolution profiles were also compared with the similarity factor (f2) test and regarded as similar when values were 50 or higher [41,42,43,44].

## 3. Results

### 3.1. Hot-Melt Extrusion

Macroscopically, the extrudates at a drug load of 20% and 25% (m/m) in either Eudragit RL or RS showed a matte, pearly structure typical of a solid solution. Very small, shiny particles in the extrudates correspond to the talcum in the formulation. The appearance of the extrudate at 33% m/m flurbiprofen differed from the other extrudates. Its cloudy appearance resembled a solid suspension, and the mechanical flexibility of this extrudate was higher than the other extrudates.

For further investigations, extrudates with a 25% m/m flurbiprofen loading were chosen as a compromise between improving mechanical flexibility and achieving the highest possible API concentration corresponding to a solid solution rather than a solid suspension. Although the mechanical flexibility of the pilot 25% m/m extrudate was acceptable, it was still too hard and brittle for further processing. To overcome this problem, 3% m/m magnesium stearate was added to the formulation as a plasticizer. Even so, the reformulated extrudate was still too brittle. For this reason, stearic acid 4% m/m was substituted for magnesium stearate (see Table 1).

As in the pilot studies, both the Eudragit RL and RS optimized extrudates showed a matte, pearly structure with very small, shiny talcum particles and, in the case of the Eudragit RS extrudates, an additional slightly yellow hue. For both Eudragit RL and RS extrudates, the addition of the plasticizer resulted in improved mechanical flexibility, even at higher API contents. The change in plasticizer may have also increased the solubility of flurbiprofen in Eudragit RS, as the solid suspension issue that had been observed in the pilot studies at 33% m/m API was not observed in the optimized Eudragit RS formulation at 35% m/m.

### 3.2. Content Uniformity

Results for content homogeneity of the optimized flurbiprofen extrudates are shown in Table 6. The results indicate excellent content uniformity of the extrudates and reproducibility of the hot-melt extrusion process, as evidenced by the low relative SD (<2%). The only exception was the results for the pilot study batch at 33% flurbiprofen, which showed a low API content, around 93% of the theoretical value. This may have been due to a weighing error, as it was consistent over all segments of the extrudate.

### 3.3. DSC

**Figure 1:**
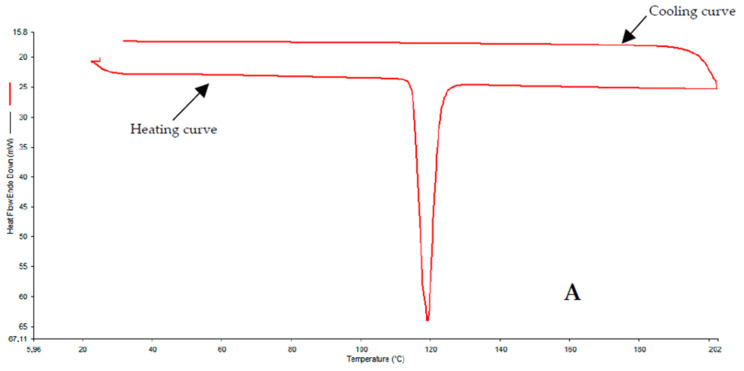

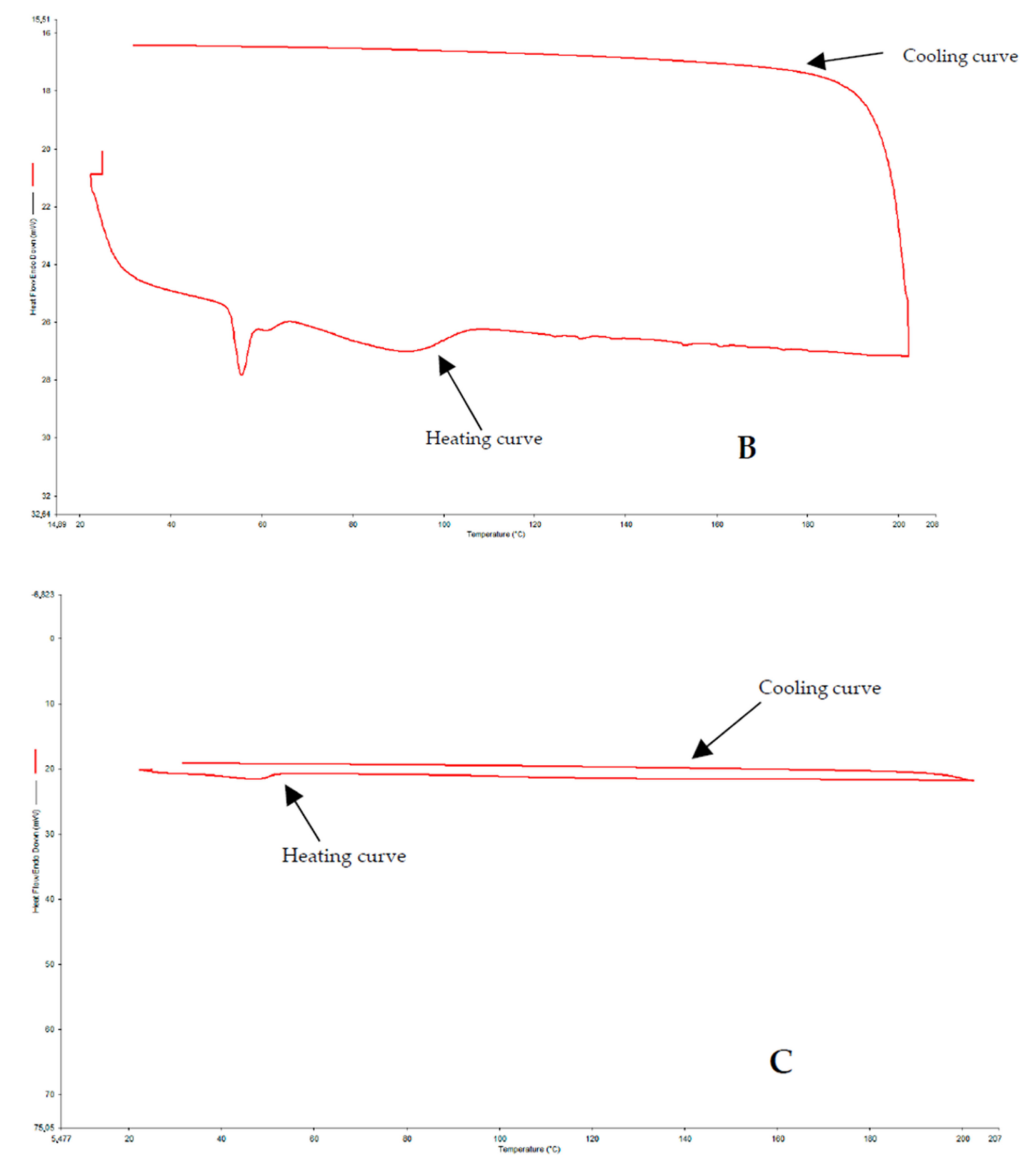
DSC thermograms for pure flurbiprofen (**A**), the physical mixture of the API with excipients (**B**) and the extrudate of the API with excipients (**C**).

Typical DSC thermograms for pure flurbiprofen, the physical mixture of the API with excipients and the extrudates are shown in Figure 1. A complete set of DSC thermograms can be found in the Supplementary Materials. Physical mixtures showed small melting peaks of flurbiprofen (at ~120 °C), Eudragit RL PO/RS PO and stearic acid (at ~55 °C). None of the extrudates showed melting peaks in the DSC corresponding to flurbiprofen, suggesting that the API was completely dissolved in the polymer. The Eudragit RS extrudates with a 30% or 35% drug load (m/m) and all the Eudragit RL extrudates showed a small peak around 50 °C, which can likely be assigned to talcum.

### 3.4. X-ray Powder Diffraction Analysis

**Figure 2:**
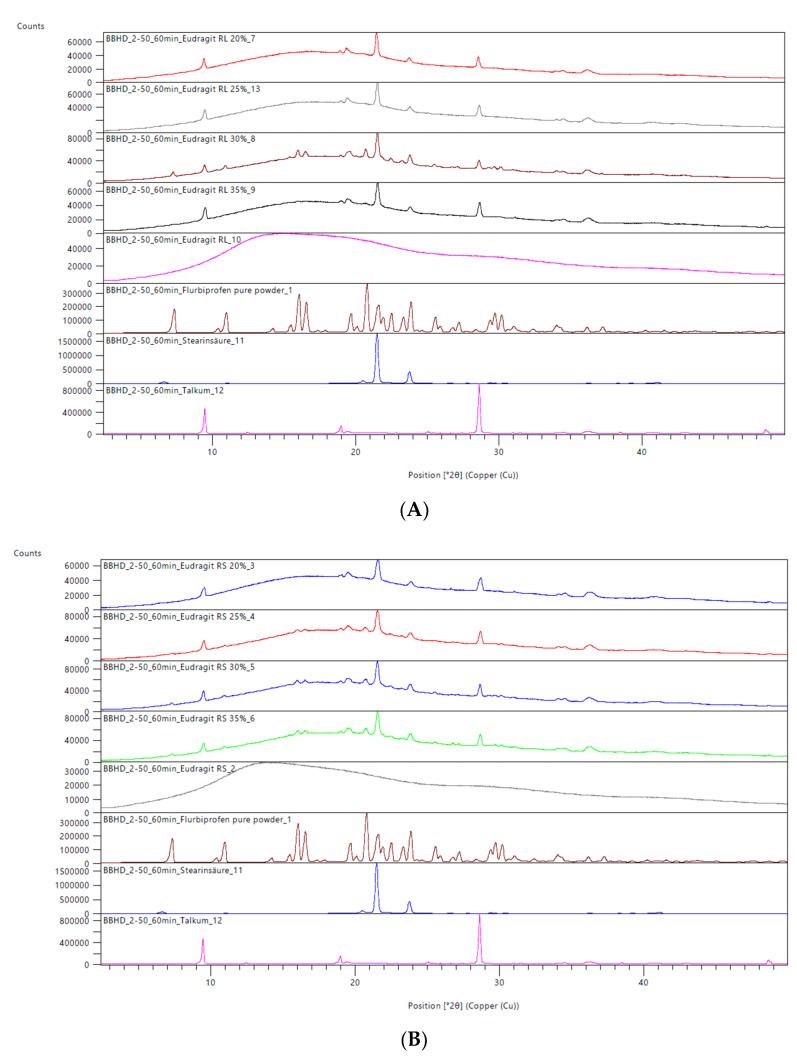
X-ray powder analysis of pulverized Eudragit RL (Panel (**A**)) and Eudragit RS extrudates (Panel (**B**)) with a flurbiprofen load between 20–35% (m/m) in comparison with the individual components.

The X-ray powder diffraction results are shown in Figure 2.

The XRPD analysis showed somewhat different results compared to the DSC analysis. Talcum showed two peaks at positions 9.5 and 28.5, with an additional small peak at position 19. Stearic acid showed peaks at positions 21.5 and 24. All these excipient peaks can also be found in the analysis of the extrudates for both Eudragit RL and RS extrudates, suggesting that talcum and stearic acid are not dissolved in the polymers. The Eudragit RL extrudate at a 30% drug load and the Eudragit RS extrudates at 25, 30 and 35% drug loads showed 4 additional small peaks at positions 7, 11 and 16, which are also visible in the analysis of pure flurbiprofen. These peaks can be attributed to small amounts of crystalline flurbiprofen that remained undissolved during the extrusion process. As XRPD is more sensitive than DSC, it is reasonable to assume that these minor amounts of crystalline flurbiprofen were not visible in the DSC analysis.

### 3.5. Dissolution Testing

The dissolution profiles from the various Eudragit RL and RS extrudate samples are shown in Figure 3, Figure 4, Figure 5, Figure 6, Figure 7, Figure 8, Figure 9 and Figure 10. All data are tabulated in the Supplementary Materials.

#### 3.5.1. Dissolution Testing of the Eudragit RL Extrudates: Drug Loading Effect

**Figure 3:**
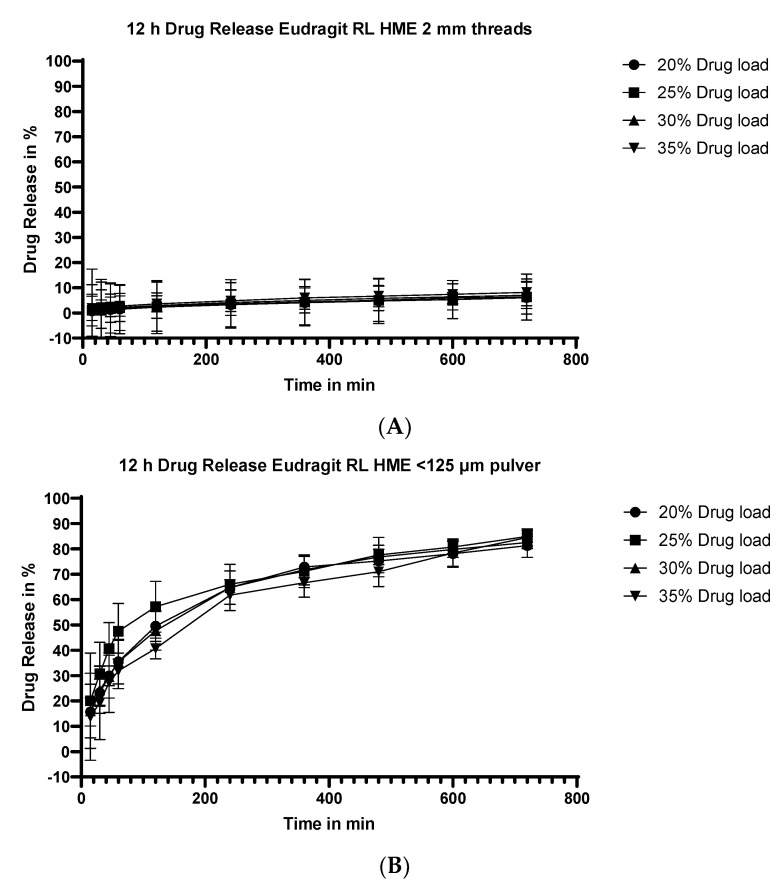
Dissolution profiles of Eudragit RL/flurbiprofen HME at different drug loads. Results for unmilled extrudate threads are shown in the upper panel (**A**), and results for the milled extrudates (particle size <125 µm) are shown in the lower panel (**B**).

Figure 3 shows the release of flurbiprofen from Eudragit RL unmilled extrudate threads (Figure 3A), and extrudates pulverized to a particle size of <125µm (Figure 3B) at various flurbiprofen payloads. At both particle sizes, there was no appreciable effect of drug loading on the percentage released as a function of time.

#### 3.5.2. Dissolution Testing of the Eudragit RL Extrudates: Effect of Particle Size

**Figure 4:**
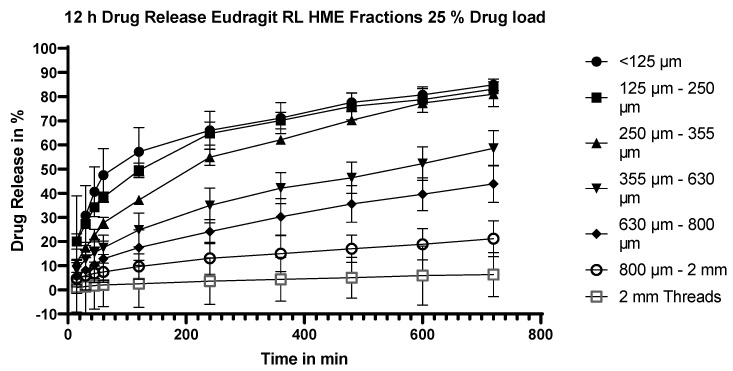
Dissolution profiles of the fractionated Eudragit RL/flurbiprofen HME extrudates at 25% m/m drug load.

Extrudates manufactured with Eudragit RL at a flurbiprofen payload of 25% were milled to produce fractions of different particle sizes, ranging from 2 mm unmilled threads to a particle size fraction with particles <125 mm in diameter. The results of dissolution testing with these fractions are shown in Figure 4. The dependency of dissolution rate and extent on the particle size is clearly demonstrated and was observed at each drug loading.

#### 3.5.3. Dissolution Testing of the Eudragit RL Extrudates: Effect of Stirring Rate

**Figure 5:**
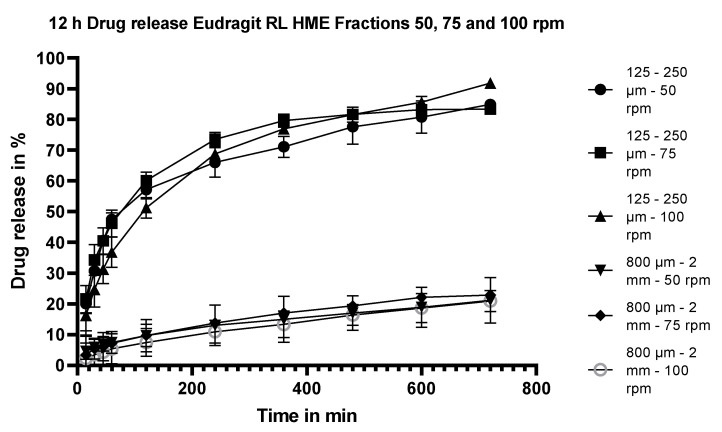
Dissolution performance of two fractions of the fractionated Eudragit RL/flurbiprofen HME extrudates (125–250 µm and 800 µm–2 mm) at 25% m/m drug load stirred at 50, 75 and 100 rpm.

Dissolution testing of milled HME extrudates (125–250 mm fraction and 800–2000 µm fraction) of flurbiprofen manufactured with Eudragit RL as the sustained-release polymer at 50, 75 and 100 rpm are shown in Figure 5. The stirring rate appeared to have little impact on the rate or extent of flurbiprofen release.

#### 3.5.4. Dissolution Testing of the Eudragit RS Extrudates: Effect of Drug Load

**Figure 6:**
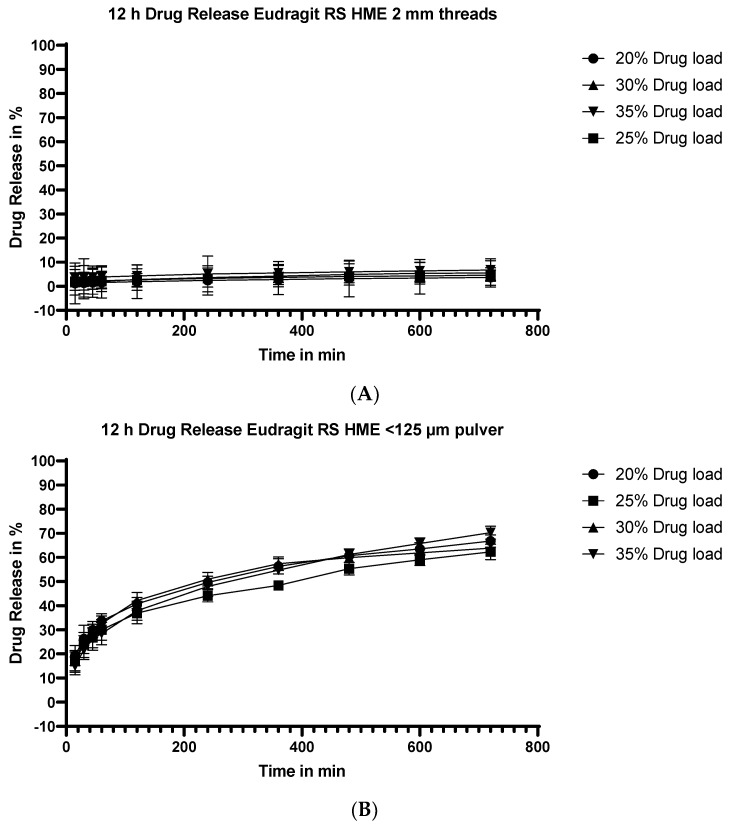
Dissolution profiles of Eudragit RS/flurbiprofen HME at different drug loads. Results for unmilled extrudate threads are shown in the upper panel (**A**), and results for milled extrudates (particle size <125µm) are shown in the lower panel (**B**).

The extrudates made with Eudragit RS were subjected to similar testing as those made with Eudragit RL. In Figure 6, the results for flurbiprofen release over 12 h as a function of flurbiprofen payload are shown for two different particle sizes of extrudates. The release from unmilled threads is shown in the upper panel (A), while the release from finely milled extrudates (particle size <125 µm) is shown in the lower panel (B).

As for extrudates prepared with Eudragit RL, the flurbiprofen payload has no appreciable impact on the percentage release as a function of time at either particle size.

#### 3.5.5. Dissolution Testing of the Eudragit RS Extrudates: Effect of Particle Size

**Figure 7:**
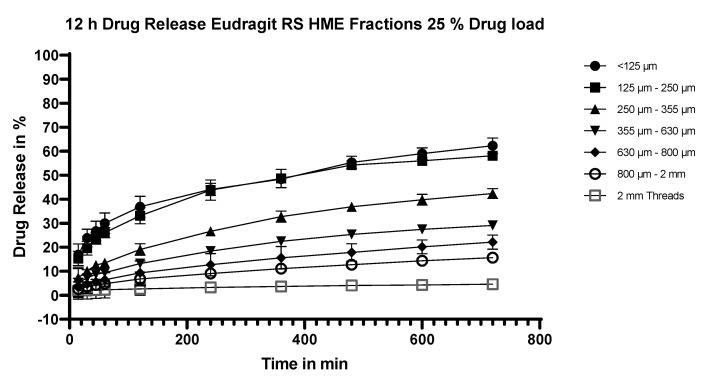
Dissolution profiles of fractionated Eudragit RS/flurbiprofen HME extrudates at 25% m/m drug load.

The effect of particle size on release from extrudates made with Eudragit RS is shown in Figure 7. For these experiments, extrudates with a 25% m/m payload were studied. There is clearly a dependency of release rate on particle size. In comparison with the results for extrudates made with Eudragit RL (Figure 4), release rates are slower at each particle size fraction.

#### 3.5.6. Dissolution Testing of the Eudragit RS Extrudates: Effect of Stirring Rate

**Figure 8:**
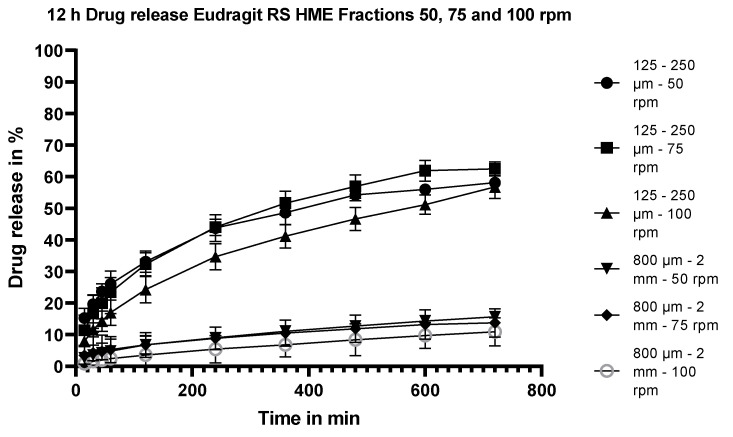
Dissolution profiles of two fractions of the fractionated Eudragit RS/flurbiprofen HME extrudates (125–250 µm and 800 mm–2 mm) at 25% m/m drug load when stirred at 50, 75 or 100 rpm.

Dissolution testing of milled HME extrudates (125–250 mm fraction and 800–2000 µm fraction) of flurbiprofen manufactured with Eudragit RS as the sustained-release polymer at 50, 75 and 100 rpm are shown in Figure 8. As for the Eudragit RL extrudates, the stirring rate of the paddle has little effect on the release profile.

#### 3.5.7. Dissolution Testing of the Commercial Product, Cebutid LP

**Figure 9:**
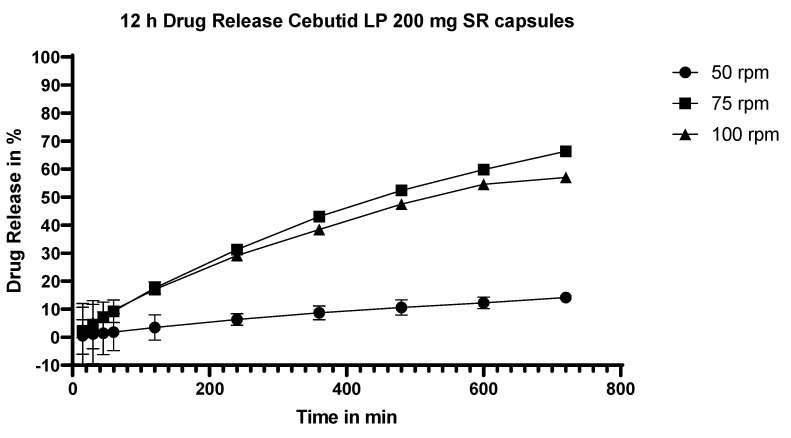
Dissolution profiles of the commercial sustained-release product when stirred at 50, 75 or 100 rpm.

In addition to testing the various exudates of flurbiprofen, the dissolution of the commercial product, Cebutid LP, was also studied, and the results are shown in Figure 9. In contrast to the exudates, the dissolution of flurbiprofen was quite dependent on the stirring rate, with a large difference between results at 50 and 75/100 rpm.

## 4. Discussion

Hot-melt extrusion is mostly used to form solid drug solutions of poorly soluble drugs with the aim of improving their dissolution and solubility characteristics and, thus, their bioavailability after oral administration [45,46,47,48,49,50]. Far less studied is the application of hot-melt extrusion with the aim of sustaining the release of the API over several hours to prolong the dosing interval [3,51,52,53,54,55,56,57].

In this work, we formulated a well-known NSAID, flurbiprofen, using hot-melt extrusion with either Eudragit RL or RS in order to better understand the role of key parameters on sustained-release performance. These parameters include drug loading, polymer type, milling and hydrodynamics. The release profile of a selected extrudate was also compared with that of a currently marketed sustained-release product containing flurbiprofen (Cebutid LP©).

The hot-melt extrusion process was unproblematic in terms of the absence of de-mixing in the extruder, achieving an optically clear extrudate and being able to mill the extrudate easily. We were able to formulate and produce flurbiprofen-loaded hot-melt extrudate threads under standard processing conditions, with only minor adaptations. The excellent recovery and content uniformity results confirmed the reproducible processability of the formulations. The recovery rate of flurbiprofen during our content uniformity investigations was around 100%, with the sole exception of the formulation containing 33% (m/m) flurbiprofen, which had a recovery rate of approx. 93%. A relative standard deviation of <2% for all tested different compositions indicated excellent content uniformity in the extrudates.

### 4.1. DSC and XRPD

In the DSC analysis, no melting peaks of the API could be observed, suggesting that all of the API was dissolved in the polymer. However, the more sensitive XRPD analysis revealed small amounts of undissolved API in Eudragit RS extrudates with a drug load of 25% (m/m) and higher. Likewise, with the exception of the Eudragit RL 30% extrudate, no undissolved API was observed in the extrudates. For the 30% extrudate, the XRPD showed some undissolved API which was traced back to an inaccuracy in the process flow. Thus, it can be concluded from the DSC and XRPD analysis that the greater part of the flurbiprofen was dissolved in the Eudragit polymer during the extrusion process and is in an amorphous state in the extrudate.

### 4.2. Loading Capacity

The maximum drug load capacity of Eudragit RS for flurbiprofen seems to be around 25% (m/m) drug load, while the maximum drug load capacity of Eudragit RL for flurbiprofen appears to be higher than 35% (m/m). In a solid dispersion, the interaction between polymer and API mainly proceeds through ionic interactions, van der Waals forces and/or hydrogen bonds [58]. As the amount of ammonium groups in Eudragit RL is twice as high as in Eudragit RS, more interactions between the API and Eudragit RL are possible, which may explain the higher drug load capacity of Eudragit RL.

### 4.3. Dissolution Testing

All extrudate formulations exhibited dissolution profiles typical for a sustained-release dosage form and commensurate with diffusion theory. The extrudates showed an initial burst effect in the dissolution profile, which appeared to be higher at lower particle sizes (see Table 7). Thereafter, they released flurbiprofen in a sustained release pattern over a period of at least 12 h. After 12 h, the extrudates were swollen but still intact. Although Eudragit RL and RS are non-erodible polymers, they do swell slightly with time, which increases the diffusivity of the API through the matrix [2].

#### 4.3.1. Effect of Particle Size

**Figure 10:**
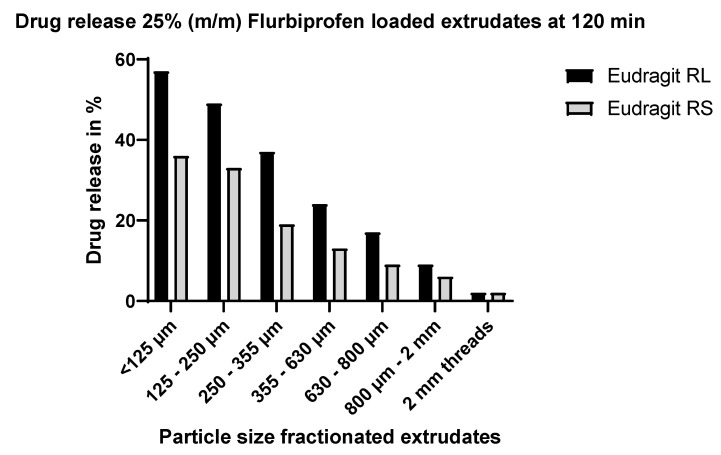
Dissolution profiles of the fractionated Eudragit RL/flurbiprofen and Eudragit RS/flurbiprofen HME extrudates at 25% (m/m) drug load at 120 min.

By milling the extrudates, the surface area and thus the diffusion of the API is increased markedly, in line with diffusion theory [18,59]. This was further investigated and confirmed by fractionating the extrudates. Milled extrudates with a particle size of <125 µm showed the highest and fastest dissolution rate for both Eudragit RL and RS extrudates at different drug loads, with a total average drug release of 83% for Eudragit RL and 66% for Eudragit RS after 12 h. This can be attributed to their large surface area per gram. At the opposite end of the scale, the extrudate threads showed the slowest and lowest dissolution, with an average total drug release of 7% for Eudragit RL and 5% for Eudragit RS. The dependency of release rate on the particle size appears to be somewhat less pronounced for Eudragit RS than Eudragit RL extrudates, but this is because the release from the Eudragit RS extrudates is slower in general (see Figure 10).

At all particle sizes of milled Eudragit RL and Eudragit RS extrudates, an initial burst release within the first 15 min of dissolution was observed, with a tendency for the burst release to be greater at the fractions with the lowest particle size (<125 µm and 125–255 µm, see Table 7). In addition to the larger surface area per gram of the milled materials and thus greater initial exposure of the API to the dissolution medium, the repeated milling process required to get to the lowest particle sizes (255 µm and below) may have resulted i a higher energy surface and therefore a greater driving force for dissolution. The initial burst in release was followed by approximately first-order release. The unmilled extrudate threads differed in their behavior. Whereas the Eudragit RL threads showed a small burst effect, the Eudragit RS threads showed a pronounced burst effect. Thereafter, both the RL and RS threads were released in an almost zero-order fashion.

#### 4.3.2. Effect of API Load

The effect of API load on the % release vs. time was not affected at the two particle size extremes, as shown in Figure 3A for the Eudragit RL extrudate threads and milled (particle size <125µm) extrudates Figure 3B, respectively, and in Figure 4A (extrudate threads) and Figure 4B (milled extrudates particle size <125 µm) for Eudragit RS extrudates. The results suggest that, as long as the greater part of the API is molecularly dispersed in the extrudate, the release rate will be independent of the drug loading. A higher drug loading is advantageous in terms of formulation, especially for APIs dosed at a higher mg dose, since it minimizes the number of units required for a given dose.

#### 4.3.3. Effect of Polymer Type

The release of flurbiprofen from Eudragit RS extrudates was lower than that from the Eudragit RL extrudates at the same API load and particle size, in accordance with the known greater sustained effect of Eudragit RS. Eudragit RL and RS have different amounts of ammonium groups (10 vs. 5%) in their chemical structure [59]. These groups are ionized in the presence of an aqueous solution, enabling the polymer to swell and become more permeable to the dissolution medium and solutes.

#### 4.3.4. Effect of Stirring Speed on Release from Extrudates

The dissolution profiles of Fraction 2 (800 µm–2 mm) and Fraction 6 (125–250 µm) of both the Eudragit RL and RS extrudates at a drug load of 25% m/m were compared at stirring speeds of 50, 75 rpm and 100 rpm. For the Eudragit RL extrudates, the drug release was not affected by the stirring speed at either particle size (see Figure 5). The drug release of the Eudragit RS extrudates was generally similar among the three stirring rates at both particle sizes (see Figure 8). Although visually, there was a slight trend for slower release at 100 rpm than at 50 or 75 rpm, an f2 test comparing the dissolution at 75 and 100 rpm resulted in an f2 value of 76 for fraction 2 with a particle size of 800 µm–2 mm and an f2 value of 56 for fraction 6 with particle size 125–250 µm, confirming similarity of the profiles. The lack of dependency of the release rate on the dissolution hydrodynamics suggests that the in vivo performance of the extrudates is unlikely to be influenced to a great extent by fluctuations in GI hydrodynamics.

#### 4.3.5. Commercial Product vs. Extrudates

**Figure 11:**
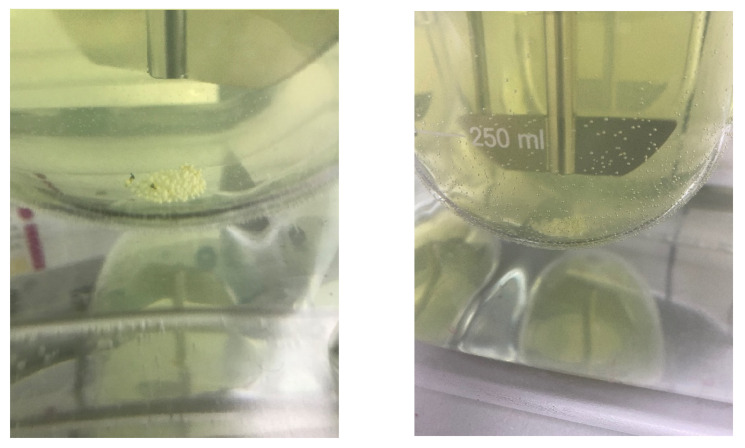
Sustained-release pellets from the commercial product at the bottom of the vessel (paddle rotation speed: 50 rpm (**left**); 75 rpm (**right**)).

The commercial product showed a slower dissolution rate compared to either Eudragit RL or RS fractions at the same particle size (the particle size of the pellets in the commercial product had a particle size range between 800 µm–1 mm) at a stirring speed of 50 rpm. After being released from the capsule, the pellets sank to the bottom of the vessel and remained there with a tendency to cone (Figure 11). For this reason, the dissolution test of the commercial product was repeated at a higher stirring speed (75 rpm and 100 rpm) to determine whether the coning phenomenon was responsible for the sluggish dissolution performance of the commercial product (Figure 9). The dissolution rate of the commercial product at 75 rpm was nearly four-fold higher than at 50 rpm. At 75 and 100 rpm, more of the pellets moved about in the dissolution medium (the cone was smaller by a factor of ~2 both in width and height), and thus more of the pellet surface was exposed to the dissolution medium.

By contrast, none of the extrudates, regardless of particle size, showed any coning behavior. Since the dissolution performance of the extrudate formulations of flurbiprofen was consistent and largely unaffected by different stirring speeds, it appears that the robustness of the extrudates against the mechanical and hydrodynamic variations in the GI tract may be higher than for the commercial product.

Taken together, the ease of processing, content uniformity, loading capacity, XRPD and dissolution results suggests a promising basis for the application of hot-melt extrusion to oral sustained-release dosage form development.

## 5. Conclusions

Using hot-melt extrusion with sustained-release polymers that are not ionizable, in this case, Eudragit RL and RS offer the ability to tailor the release of the API to the therapeutic indication through a combination of particle size and polymer choice while providing robustness over a range of hydrodynamic conditions. The results from this study confirm the ability to adapt the dissolution performance of sustained-release hot-melt extrudates by changing the particle size (and hence the surface area) [58,59,60,61] or modifying the composition of the extrudates [2,3,35,36].

This flexibility opens up a wide variety of possibilities for different applications in the treatment of inflammatory diseases. The timespan of drug release from the extrudate formulation can be adjusted by modifying the drug release from the extrudate formulation. For example, for the therapy of Crohn’s disease, where intermittent areas of inflammation are found along the GIT [62], slow release over a long period starting with entry into the small intestine, would be desirable. For the therapy of rheumatoid arthritis [14,15], a prolonged release initiated soon after ingestion and continuing for 8–12 h may facilitate twice daily dosing while still ensuring 24/7 coverage. Since the currently recommended dosing schedule is up to four times per day for immediate-release products containing flurbiprofen [63], sustained-release formulations would represent better and more convenient therapy.

## Figures and Tables

**Table 1:** Extrusion-relevant properties of the drug and polymers.

	Flurbiprofen	Eudragit RL	Eudragit RS
Melting temperature	110–111 °C ^a^ 114–118 °C ^b^	-	-
Glass transition temperature	−8.15 °C	63 °C	64 °C
Degradation temperature	184 °C	166 °C	170 °C

^a^ Literature values for the pure S enantiomer of flurbiprofen [30,31,32]; ^b^ Literature values for the racemate of flurbiprofen [33,34,35]. In these studies, we used the racemate.

**Table 2:** Qualitative and quantitative composition of the flurbiprofen extrudates in the pilot studies.

Components	20% Flurbiprofen	25% Flurbiprofen	33% Flurbiprofen	25% Flurbiprofen	Function
Flurbiprofen	20%	25%	33%	25%	API
Talcum	2.5%	2.5%	2.5%	4%	Homogenization
Eudragit RS PO	75%	70%	62%	-	Polymer
Eudragit RL PO	-	-	-	67%	Polymer
Mg Stearate	2.5%	2.5%	2.5%	-	Plasticizer
Stearic acid	-	-	-	4%	Plasticizer

**Table 3:** Qualitative and quantitative composition of the optimized flurbiprofen hot-melt extrudates.

Components	20% Flurbiprofen	25% Flurbiprofen	30% Flurbiprofen	35% Flurbiprofen	Function
Flurbiprofen	20%	25%	30%	35%	API
Talcum	4%	4%	4%	4%	Homogenization
Eudragit RS PO	72%	67%	62%	57%	Polymer
Stearic acid	4%	4%	4%	4%	Plasticizer

**Table 4:** Particle size range of each fraction of the milled extrudates containing 25% m/m flurbiprofen.

Fraction	Particle Size Fraction
1	2 mm threads
2	800 µm–2 mm
3	630 µm–800 µm
4	355 µm–630 µm
5	250 µm–355 µm
6	125 µm–250 µm
7	<125 µm

**Table 5:** HPLC Parameters for the analysis of flurbiprofen content and dissolution in the optimized hot-melt extrudates.

Parameter	Value
Mobile phase and pH	ACN: H_2_O: TFA 58:42:0.1 pH 6.5
Flow rate	1.0 mL/min
Absorption wavelength	248 nm
Retention time	8.8 min
Correlation coefficient	0.999
LOQ	2.26 µg/mL
Method reference	Nothnagel and Jung [40]

**Table 6:** Percentage of target flurbiprofen content in the various segments of the optimized extrudate threads (*n* = 3 per segment in each batch).

Flurbiprofen % Loading (m/m)	Start	Middle	End	Mean	Rel.SD
**E-RL extrudates**					
**20%**					
1. Batch	99.19%	100.89%	99.56%	99.88%	0.89%
2. Batch	101.63%	99.74%	100.24%	100.54%	0.97%
**25%**					
1. Batch	101.53%	100.65%	100.22%	100.80%	0.67%
2. Batch	99.67%	100.61%	99.27%	100.15%	0.66%
**30%**					
1. Batch	100.71%	100.67%	99.55%	100.31%	0.66%
2. Batch	100.25%	99.29%	99.47%	99.67%	0.68%
**35%**					
1. Batch	100.41%	99.81%	99.19%	99.81%	0.61%
2. Batch	100.21%	101.47%	100.04%	100.58%	0.89%
**E-RS extrudates**					
**20%**					
1. Batch	101.92%	99.51%	99.18%	100.21%	1.49%
2. Batch	101.47%	99.35%	98.95%	99.93%	1.50%
**25%**					
1. Batch	99.93%	100.21%	101.73%	100.63%	0.97%
2. Batch	100.17%	101.09%	98.63%	100.64%	0.65%
**30%**					
1. Batch	99.43%	99.21%	97.97%	98.87%	0.80%
2. Batch	99.88%	100.98%	99.89%	100.44%	0.77%
**35%**					
1. Batch	101.60%	99.76%	98.84%	100.07%	1.41%
2. Batch	99.39%	101.74%	99.53%	100.57%	1.65%

**Table 7:** Burst release from the fractionated Eudragit RL and RS extrudates at 25% m/m drug load.

Burst Release from the Fractionated Eudragit RL Extrudates at 25% (/m/m) Drug Load							
Fractions	<125 µm	125–250 µm	250–355 µm	355–630 µm	630–800 µm	800 µm–2 mm	2 mm Threads
Drug release (%) at 15 min	20.10	20.04	11.46	8.28	5.58	4.64	0.96
Drug release (%) at 720 min	84.90	83.14	81.01	58.57	43.87	21.18	6.30
Burst Release in %	23.6	24.1	14.1	14.1	12.7	21.9	15.2
Burst release from the fractionated Eudragit RS extrudates at 25% (/m/m) drug load							
Drug release (%) at 15 min	16.92	15.27	7.34	5.26	3.26	2.66	1.98
Drug release (%) at 720 min	62.26	58.09	42.38	29.06	22.13	15.67	4.59
Burst Release in %	26.8	26.2	17.3	18.1	14.7	16.9	43.1

## Data Availability

Not applicable.

## Supplementary Materials

The following supporting information can be downloaded at: https://www.mdpi.com/article/10.3390/pharmaceutics15071996/s1. Table S1: Percentage of target flurbiprofen content in the different segments of the extrudate threads from the pilot studies (*n* = 3); Table S2. Dissolution performance of the Eudragit RL/flurbiprofen HME extrudates threads at different drug loads; Table S3. Dissolution performance of the pulverized (particle size <125 µm) Eudragit RL/flurbiprofen HME extrudates at different drug loads; Table S4. Dissolution performance of the fractionated Eudragit RL/flurbiprofen HME extrudates at 25% (m/m) drug load; Table S5. Dissolution performance of 125–250 µm fraction of the fractionated Eudragit RL/flurbiprofen HME extrudates at 25% (m/m) drug load at 50, 75 and 100 rpm; Table S6. Dissolution performance of 800 µm–2 mm fraction of the fractionated Eudragit RL/flurbiprofen HME extrudates at 25% (m/m) drug load at 50, 75 and 100 rpm; Table S7. Dissolution performance of the Eudragit RS/flurbiprofen HME extrudate threads at different drug loads; Table S8. Dissolution performance of the Eudragit RS/flurbiprofen HME extrudate threads at different drug loads; Table S9. Dissolution performance of the pulverized Eudragit RS/flurbiprofen HME extrudates (particle size < 125 µm) at different drug load; Table S10. Dissolution performance of the fractionated Eudragit RS/flurbiprofen HME extrudates at 25% (m/m) drug load; Table S11. Dissolution performance of 125–250 µm fraction of the fractionated Eudragit RS/flurbiprofen HME extrudates at 25% (m/m) drug load stirred at 50, 75 and 100 rpm; Table S12. Dissolution performance of the 800 µm–2 mm fraction of the fractionated Eudragit RS/flurbiprofen HME extrudates at 25% (m/m) drug load stirred at 50, 75 and 100 rpm; Table S13. Dissolution performance of the commercial sustained release product at stirring rates of 50, 75 and 100 rpm; Figure S1. DSC of pure flurbiprofen, pure excipients, physical mixtures and extrudates.
