# Supplementary material for: Factors That Influence Sustained Release from Hot-Melt Extrudates
Source: Pharmaceutics. 2023 Jul 20;15(7):1996. doi: 10.3390/pharmaceutics15071996 (PMC10386192; doi:10.3390/pharmaceutics15071996)
Supplement: Supplementary file 1 [file pharmaceutics-15-01996-s001.zip › pharmaceutics-2424305-supplementary.pdf]

Factors which influence sustained release from hot-melt extrudates

Supplementary Material

TABLES

Table S1 Percentage of target flurbiprofen content in the different segments of the extrudate threads from the pilot studies (n=3)

Flurbiprofen conc. (m/m)	Start	Middle	End	Mean	Rel. SD
20%	100.75%	101.53%	101.61%	101.3%	0.38%
25%	102.08%	101.77%	100.32%	101.39%	0.75%
33%	92.98%	93.05%	93.03%	93.02%	0.03%
25% + 3% Mg-Stearate	103.70%	103.86%	103.09%	103.55%	0.003%
25% with Eudragit RL PO + 4% stearic acid	101.13%	99.76%	100.08%	100.32%	0.71%

Results for Eudragit RL extrudates

Table S2. Dissolution performance of the Eudragit RL/flurbiprofen HME extrudates threads at different drug loads

Eudragit RL/flurbiprofen extrduate threads at different drug loads								
**Drug Load**	**20% (m/m)**		**25% (m/m)**		**30% (m/m)**		**35% (m/m)**	
**Time in min**	Release in %	Rel STD	Release in %	Rel STD	Release in %	Rel STD	Release in %	Rel STD
**15**	0.76	16.68	0.98	10.28	1.15	6.25	1.81	4.88
**30**	0.97	11.27	1.33	12.01	1.57	7.64	2.15	3.00
**45**	1.26	10.75	1.75	9.75	1.87	5.61	2.47	3.91
**60**	1.49	9.78	2.02	8.99	2.15	5.58	2.71	4.00
**120**	2.27	10.55	2.51	9.78	2.89	5.07	3.59	3.23
**240**	3.25	8.77	3.58	9.60	4.12	5.15	4.83	4.25
**360**	4.07	9.15	4.33	9.08	4.97	4.98	5.99	4.50
**480**	4.76	8.99	5.01	8.49	5.78	4.84	6.68	4.26
**600**	5.33	7.61	5.91	2.23	6.38	5.22	7.44	4.24
**720**	6.08	6.50	6.30	9.14	7.03	5.27	8.13	5.32

Table S3. Dissolution performance of the pulverized (particle size <125 µm) Eudragit RL/flurbiprofen HME extrudates at different drug loads

Eudragit RL/flurbiprofen extrduate pulverized (particle size <125 µm) at different drug load								
**Drug Load**	**20% (m/m)**		**25% (m/m)**		**30% (m/m)**		**35% (m/m)**	
**Time in min**	Release in %	Rel STD	Release in %	Rel STD	Release in %	Rel STD	Release in %	Rel STD
**15**	15.66	5.62	20.10	18.83	16.03	10.61	13.77	17.22
**30**	23.22	5.30	30.73	12.41	24.38	9.24	19.26	14.56
**45**	29.89	3.87	40.63	10.32	29.56	8.47	27.28	11.83
**60**	35.52	8.91	47.46	11.02	35.36	8.57	31.89	7.00
**120**	49.52	6.08	57.16	10.00	47.69	7.62	40.70	4.11
**240**	64.70	1.37	66.03	7.89	64.75	6.62	61.74	6.08
**360**	72.86	4.20	71.11	6.41	71.73	5.93	66.65	5.77
**480**	75.20	4.64	77.61	3.83	76.77	7.78	70.99	5.86
**600**	78.09	4.95	80.78	2.59	79.79	3.16	78.37	5.58
**720**	81.27	4.63	84.90	2.38	82.52	2.41	84.48	3.45

Table S4. Dissolution performance of the fractionated Eudragit RL/flurbiprofen HME extrudates at 25% (m/m) drug load

Fractionated Eudragit RL/flurbiprofen extrudate at 25% (m/m) drug load														
**Drug Load**	**< 125 µm**		**125 µm – 250 µm**		**250 µm – 355 µm**		**355 µm – 630 µm**		**630 µm – 800 µm**		**800 µm – 2 mm**		**2 mm threads**	
**Time in min**	Release in %	Rel STD	Release in %	Rel STD	Release in %	Rel STD	Release in %	Rel STD	Release in %	Rel STD	Release in %	Rel STD	Release in %	Rel STD
**15**	20.10	18.83	20.04	3.21	11.46	0.93	8.28	3.91	5.58	7.10	4.64	2.62	0.98	10.28
**30**	30.73	12.41	27.28	1.21	17.42	0.92	12.59	7.10	8.15	8.16	5.93	1.12	1.33	12.01
**45**	40.63	10.32	34.21	1.74	22.44	2.60	15.37	5.54	10.35	7.40	6.78	2.13	1.75	9.75
**60**	47.46	11.02	38.34	2.14	27.73	2.25	17.46	2.83	12.85	9.74	7.42	3.59	2.02	8.99
**120**	57.16	10.00	49.44	3.03	37.18	0.30	24.78	6.98	17.53	7.37	9.68	5.29	2.51	9.78
**240**	66.03	7.89	64.76	4.78	54.92	3.31	34.98	7.28	24.11	5.00	13.05	6.60	3.58	9.60
**360**	71.11	6.41	70.14	3.41	62.14	1.28	42.08	6.46	30.26	7.61	14.96	7.48	4.33	9.08
**480**	77.61	3.83	75.99	5.62	70.23	1.57	46.42	6.50	35.59	7.57	17.02	5.63	5.01	8.49
**600**	80.78	2.59	78.76	5.31	77.34	0.58	52.29	6.99	39.58	6.82	18.89	6.50	5.91	12.23
**720**	84.90	2.38	83.14	0.69	81.01	5.10	58.57	7.32	43.87	7.72	21.18	7.43	6.30	9.14

Table S5. Dissolution performance of 125 – 250 µm fraction of the fractionated Eudragit RL/flurbiprofen HME extrudates at 25% (m/m) drug load at 50,75 and 100 rpm

**Fractionated Eudragit RL/flurbiprofen extrudate at 25% (m/m) drug load, 125 – 250 µm particle size**						
**Rpm**	**50 rpm**		**75 rpm**		**100 rpm**	
**Time in min**	Release in %	Rel STD	Release in %	Rel STD	Release in %	Rel STD
**15**	20.04	3.21	21.52	4.44	16.25	6.43
**30**	30.73	1.21	34.31	5.03	24.69	5.73
**45**	40.63	1.74	40.46	4.33	31.31	4.76
**60**	47.46	2.14	46.17	4.34	36.77	4.90
**120**	57.16	3.03	60.09	2.78	51.28	3.32
**240**	66.03	4.78	73.58	2.30	68.80	2.83
**360**	71.11	3.41	79.64	2.11	76.93	1.26
**480**	77.61	5.63	81.66	1.61	81.56	2.46
**600**	80.78	5.31	83.15	0.91	85.66	1.94
**720**	84.90	0.69	83.39	1.26	91.83	0.80

Table S6. Dissolution performance of 800 µm – 2 mm fraction of the fractionated Eudragit RL/flurbiprofen HME extrudates at 25% (m/m) drug load at 50,75 and 100 rpm

**Fractionated Eudragit RL/flurbiprofen extrudate at 25% (m/m) drug load, 800 µm–2 mm particle size**						
**Rpm**	**50 rpm**		**75 rpm**		**100 rpm**	
**Time in min**	Release in %	Rel STD	Release in %	Rel STD	Release in %	Rel STD
**15**	4.64	2.62	3.35	7.90	2.22	7.19
**30**	5.93	1.12	5.40	3.37	3.46	4.94
**45**	6.78	2.13	6.10	4.57	4.33	5.01
**60**	7.42	3.59	7.16	0.90	5.36	4.93
**120**	9.68	5.29	9.72	3.63	7.45	4.55
**240**	13.05	6.60	13.74	0.85	10.93	3.71
**360**	14.96	7.48	17.01	0.70	13.33	4.29
**480**	17.02	5.63	19.33	1.52	16.40	3.35
**600**	18.89	6.50	22.09	0.47	18.68	4.72
**720**	21.18	7.43	22.90	1.48	20.95	3.44

Table S7. Dissolution performance of the Eudragit RS/flurbiprofen HME extrudate threads at different drug loads

Eudragit RS/flurbiprofen extrduate threads at different drug load								
**Drug Load**	**20% (m/m)**		**25% (m/m)**		**30% (m/m)**		**35% (m/m)**	
**Time in min**	Release in %	Rel STD	Release in %	Rel STD	Release in %	Rel STD	Release in %	Rel STD
**15**	1.19	8.50	1.98	3.60	1.65	5.32	3.31	5.02
**30**	1.35	4.53	2.05	3.64	1.68	6.91	3.44	8.00
**45**	1.47	6.10	2.18	3.47	1.87	5.20	3.68	4.81
**60**	1.55	6.41	2.27	3.47	2.06	4.25	3.84	4.67
**120**	1.90	6.94	2.67	2.70	2.75	4.51	4.27	4.66
**240**	2.41	6.10	3.27	1.95	3.57	3.96	5.07	7.46
**360**	2.81	6.31	3.69	2.14	4.24	4.43	5.48	4.79
**480**	3.19	7.60	4.09	1.99	4.90	4.42	5.95	4.50
**600**	3.38	6.65	4.30	2.22	5.34	4.45	6.37	4.73
**720**	3.68	4.01	4.59	2.15	5.53	5.17	6.76	4.68

Results for Eudragit RS extrudates

Table S8. Dissolution performance of the Eudragit RS/flurbiprofen HME extrudate threads at different drug loads

Eudragit RS/flurbiprofen extrduate threads at different drug load								
**Drug Load**	**20% (m/m)**		**25% (m/m)**		**30% (m/m)**		**35% (m/m)**	
**Time in min**	Release in %	Rel STD	Release in %	Rel STD	Release in %	Rel STD	Release in %	Rel STD
**15**	1.19	8.50	1.98	3.60	1.65	5.32	3.31	5.02
**30**	1.35	4.53	2.05	3.64	1.68	6.91	3.44	8.00
**45**	1.47	6.10	2.18	3.47	1.87	5.20	3.68	4.81
**60**	1.55	6.41	2.27	3.47	2.06	4.25	3.84	4.67
**120**	1.90	6.94	2.67	2.70	2.75	4.51	4.27	4.66
**240**	2.41	6.10	3.27	1.95	3.57	3.96	5.07	7.46
**360**	2.81	6.31	3.69	2.14	4.24	4.43	5.48	4.79
**480**	3.19	7.60	4.09	1.99	4.90	4.42	5.95	4.50
**600**	3.38	6.65	4.30	2.22	5.34	4.45	6.37	4.73
**720**	3.68	4.01	4.59	2.15	5.53	5.17	6.76	4.68

Table S9. Dissolution performance of the pulverized Eudragit RS/flurbiprofen HME extrudates (particle size < 125 µm) at different drug load

Eudragit RS/flurbiprofen extrudate pulverized (particle size <125 µm) at different drug load								
**Drug Load**	**20% (m/m)**		**25% (m/m)**		**30% (m/m)**		**35% (m/m)**	
**Time in min**	Release in %	Rel STD	Release in %	Rel STD	Release in %	Rel STD	Release in %	Rel STD
**15**	19.28	4.19	16.92	4.54	16.86	3.92	14.99	3.66
**30**	26.19	2.66	23.84	3.65	25.13	6.65	21.65	4.02
**45**	29.87	2.41	26.70	4.17	30.13	3.36	26.08	4.54
**60**	33.65	2.92	29.94	4.32	32.59	3.13	28.40	4.63
**120**	40.71	2.68	36.86	4.39	42.07	3.35	37.90	3.96
**240**	49.60	2.56	44.05	2.54	50.94	2.81	47.95	4.23
**360**	56.28	3.11	48.40	1.83	57.34	2.84	54.72	1.71
**480**	60.68	2.84	55.31	2.59	59.86	1.90	61.16	1.38
**600**	63.49	2.62	59.01	2.41	61.84	1.15	65.77	2.32
**720**	66.76	2.53	62.26	3.20	63.83	1.97	70.28	2.66

Table S10. Dissolution performance of the fractionated Eudragit RS/flurbiprofen HME extrudates at 25% (m/m) drug load

Fractionated Eudragit RS/flurbiprofen extrudate at 25% (m/m) drug load														
**Drug Load**	**< 125 µm**		**125 µm – 250 µm**		**250 µm – 355 µm**		**355 µm – 630 µm**		**630 µm – 800 µm**		**800 µm – 2 mm**		**2 mm threads**	
**Time in min**	Release in %	Rel STD	Release in %	Rel STD	Release in %	Rel STD	Release in %	Rel STD	Release in %	Rel STD	Release in %	Rel STD	Release in %	Rel STD
**15**	16.92	4.54	15.27	3.10	7.34	3.76	5.26	6.25	3.26	3.75	2.66	1.51	1.98	3.60
**30**	23.84	3.65	19.63	2.87	10.11	1.53	7.23	3.42	4.63	3.84	3.54	1.55	2.05	3.64
**45**	26.70	4.17	23.67	2.44	12.56	1.18	8.81	2.23	5.65	3.48	4.25	1.76	2.18	3.47
**60**	29.94	4.32	26.10	2.13	13.49	0.41	9.38	0.72	6.48	2.89	4.83	1.60	2.27	3.47
**120**	36.86	4.39	33.13	3.37	19.07	2.36	13.19	1.87	9.31	4.98	6.79	2.85	2.67	2.70
**240**	44.05	2.54	43.78	4.24	26.66	1.35	18.38	1.89	12.76	4.69	9.05	0.69	3.27	1.95
**360**	48.40	1.83	48.63	3.81	32.80	2.33	22.47	1.75	15.61	4.73	11.10	1.31	3.69	2.14
**480**	55.31	2.59	54.23	1.85	36.84	1.13	25.36	0.31	17.83	3.64	12.70	1.56	4.09	1.99
**600**	59.01	2.41	56.01	1.46	39.79	2.24	27.48	1.08	20.15	2.85	14.34	1.46	4.30	2.22
**720**	62.26	3.20	58.09	1.78	42.38	2.06	29.06	0.77	22.13	2.91	15.67	1.65	4.59	2.15

Table S11. Dissolution performance of 125 – 250 µm fraction of the fractionated Eudragit RS/flurbiprofen HME extrudates at 25% (m/m) drug load stirred at 50,75 and 100 rpm

Fractionated Eudragit RS/flurbiprofen extrudate at 25% (m/m) drug load, 125 – 250 µm particle size						
**Rpm**	**50 rpm**		**75 rpm**		**100 rpm**	
**Time in min**	Release in %	Rel STD	Release in %	Rel STD	Release in %	Rel STD
**15**	15.27	3.10	11.40	4.81	7.94	1.27
**30**	19.63	2.87	16.75	5.92	11.58	2.21
**45**	23.67	2.44	19.95	4.93	14.33	3.20
**60**	26.10	2.13	23.43	6.76	16.98	4.00
**120**	33.13	3.37	32.34	3.65	24.28	4.17
**240**	43.78	4.24	44.00	2.62	34.70	4.12
**360**	48.63	3.81	51.67	3.84	41.19	3.72
**480**	54.23	1.85	56.93	3.69	46.64	3.72
**600**	56.01	1.46	61.90	3.31	51.20	3.07
**720**	58.09	1.78	62.50	2.21	56.79	3.66

Table S12. Dissolution performance of the 800 µm – 2 mm fraction of the fractionated Eudragit RS/flurbiprofen HME extrudates at 25% (m/m) drug load stirred at 50, 75 and 100 rpm

Fractionated Eudragit RL/flurbiprofen extrudate at 25% (m/m) drug load, 800 µm–2 mm particle size						
**Rpm**	**50 rpm**		**75 rpm**		**100 rpm**	
**Time in min**	Release in %	Rel STD	Release in %	Rel STD	Release in %	Rel STD
**15**	2.66	1.51	3.46	2.74	0.90	9.79
**30**	3.54	1.55	4.25	2.44	1.55	10.47
**45**	4.25	1.76	4.72	2.66	1.99	7.90
**60**	4.83	1.60	5.31	4.09	2.51	6.09
**120**	6.79	2.85	6.78	3.96	3.61	6.17
**240**	9.05	0.69	8.83	3.56	5.44	4.38
**360**	11.10	1.31	10.52	4.14	6.81	3.87
**480**	12.70	1.56	11.93	4.35	8.42	5.05
**600**	14.34	1.46	13.27	4.68	9.77	4.13
**720**	15.67	1.65	13.78	4.48	10.96	4.45

Results for the commercial product, Cebutid

Table S13. Dissolution performance of the commercial sustained release product at stirring rates of 50,75 and 100 rpm

Dissolution performance Cebutid LP sustained release capsules						
**Rpm**	**50 rpm**		**75 rpm**		**100 rpm**	
**Time in min**	Release in %	Rel STD	Release in %	Rel STD	Release in %	Rel STD
**15**	0.55	11.52	2.29	8.37	3.02	3.25
**30**	1.14	10.57	4.50	8.63	5.30	1.48
**45**	1.42	7.63	7.10	5.47	7.33	1.51
**60**	1.84	6.64	9.22	4.04	9.67	1.65
**120**	3.49	4.53	17.54	2.21	17.00	0.82
**240**	6.30	2.08	31.28	1.83	29.13	1.48
**360**	8.69	2.46	43.06	1.46	38.42	1.35
**480**	10.65	2.73	52.38	1.53	47.48	1.26
**600**	12.24	2.03	59.79	1.61	54.59	0.80
**720**	14.13	1.36	66.38	1.86	57.06	1.23

Figure S1. DSC of pure flurbiprofen, pure excipients, physical mixtures and extrudates

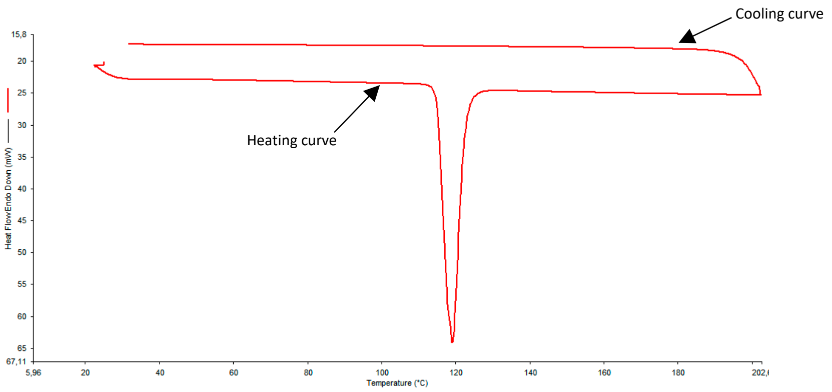

Figure S1.1 pure flurbiprofen

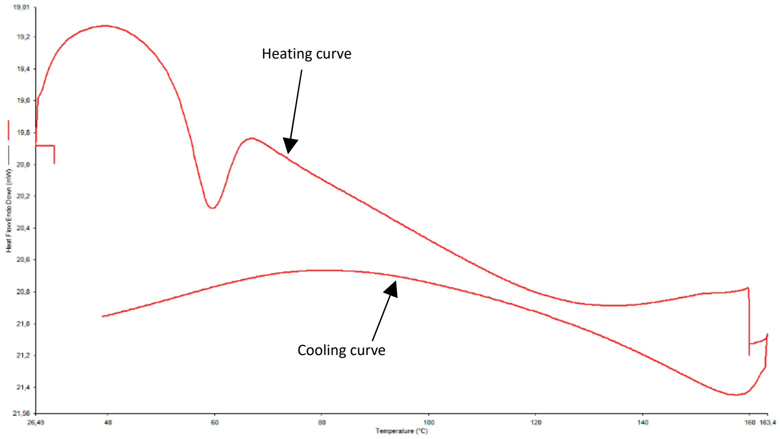

Figure S1.2. of pure talcum

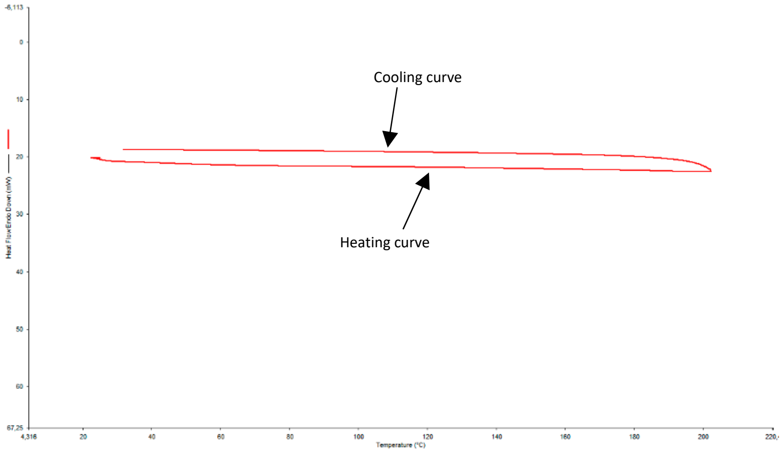

Figure S1.3. pure stearic acid

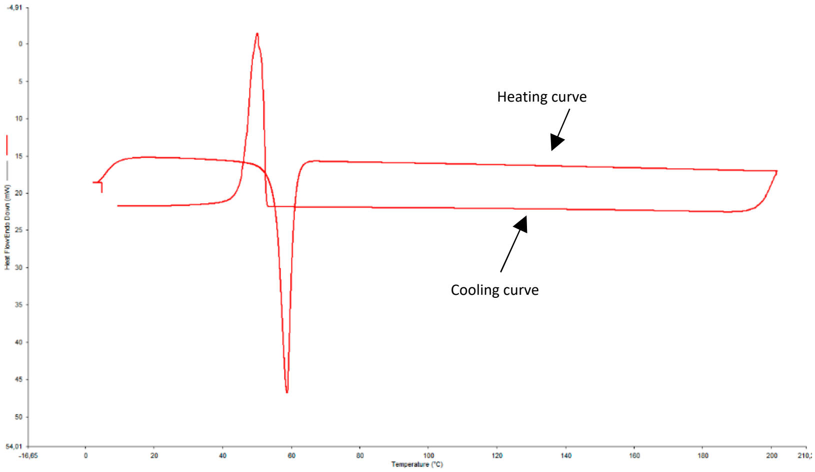

Figure S1.4. pure Eudragit RL

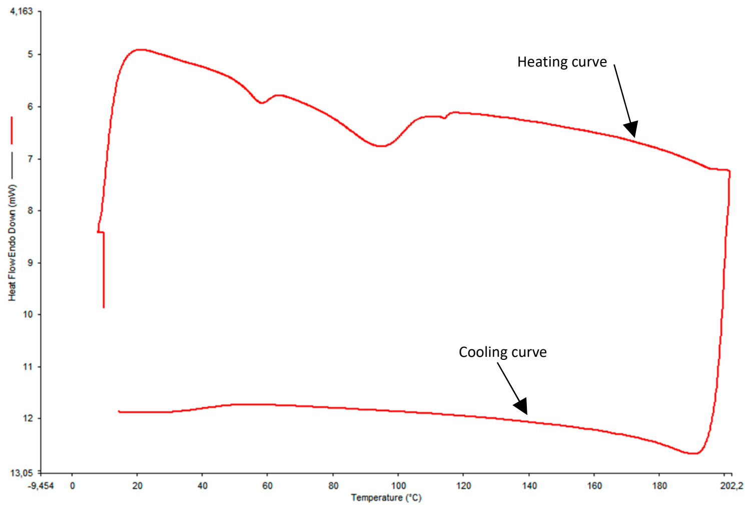

Figure S1.5 pure Eudragit RS

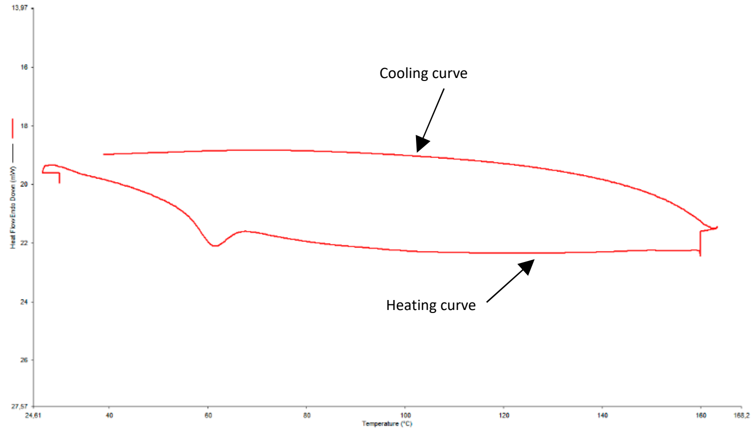

Figure S1.6 physical mixture of Eudragit RS, stearic acid and talcum containing 25% m/m flurbiprofen

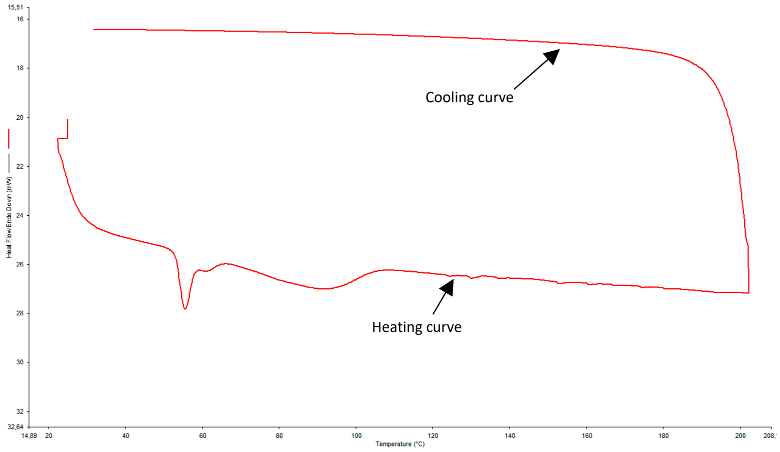

Figure S1.7. DSC thermogram of a physical mixture of Eudragit RL, stearic acid and talcum, containing 25% m/m flurbiprofen

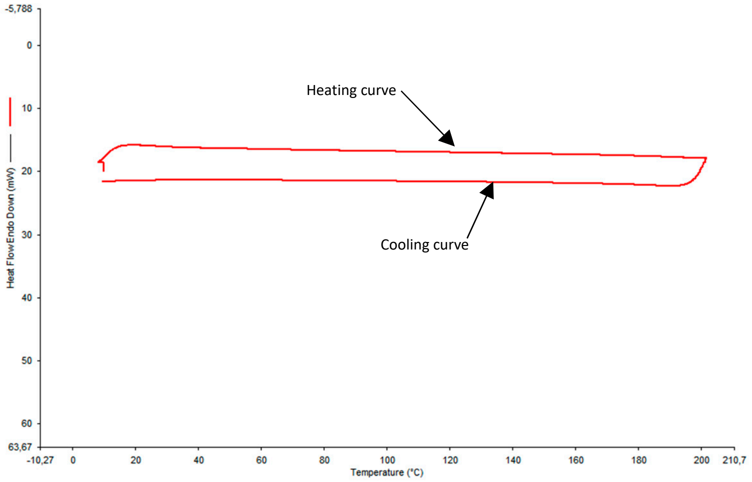

Figure S1.8. extrudate containing Eudragit RS, stearic acid, talcum and 20% m/m flurbiprofen

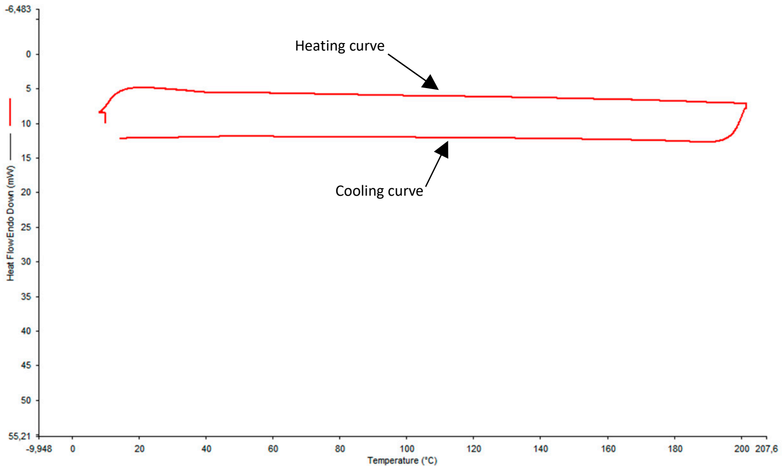

Figure S1.9 extrudate containing Eudragit RS, stearic acid, talcum and 25% m/m flurbiprofen

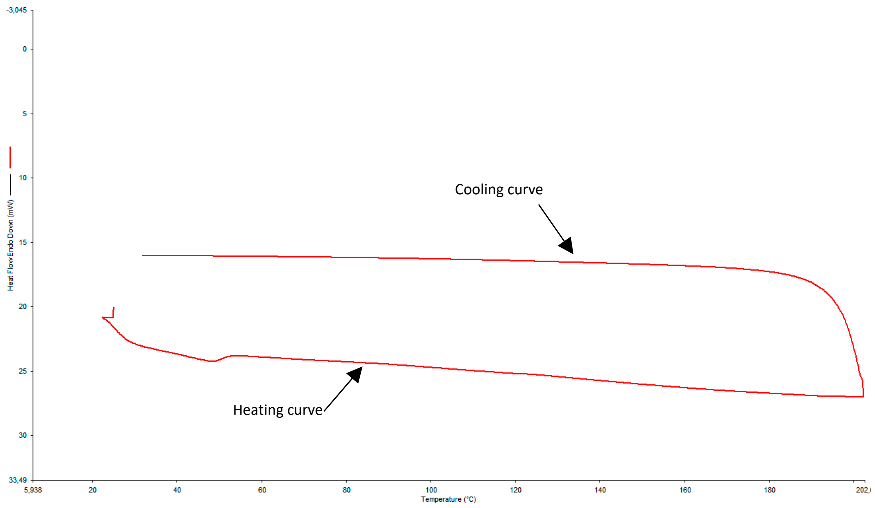

Figure S1.10. extrudate containing Eudragit RS, stearic acid, talcum and 30% m/m flurbiprofen

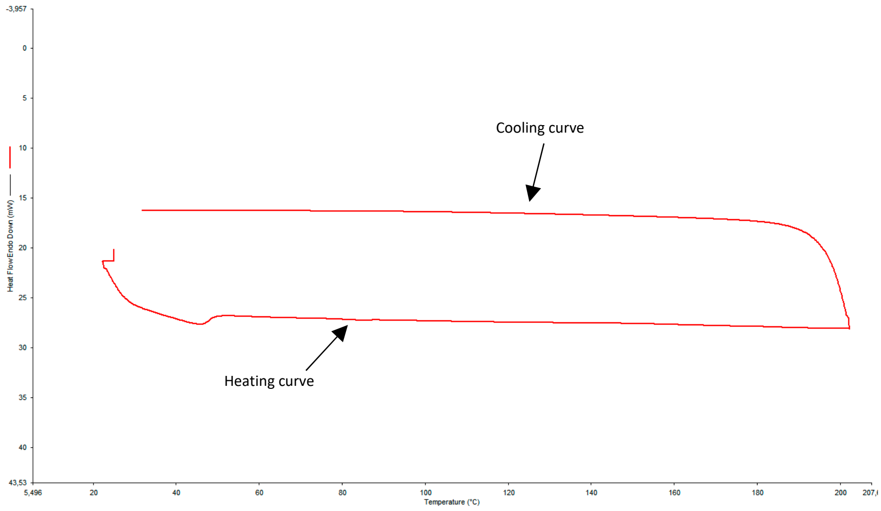

Figure S1.11. extrudate containing Eudragit RS, stearic acid, talcum and 35% m/m flurbiprofen

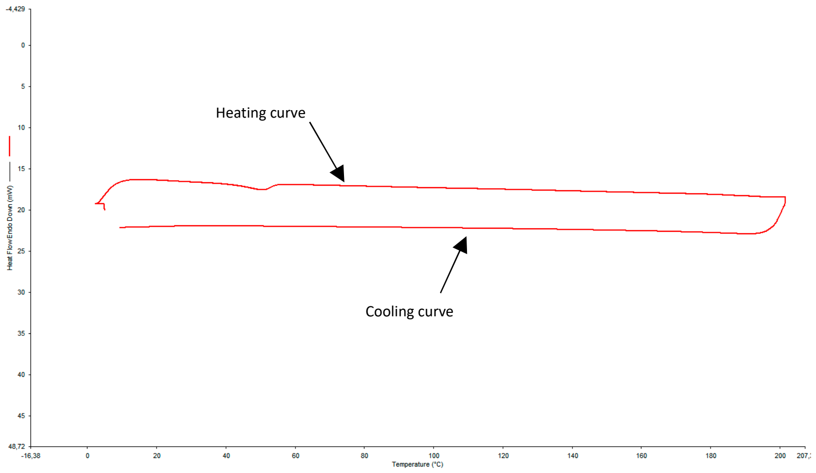

Figure S1.12. extrudate containing Eudragit RL, stearic acid, talcum and 20% m/m flurbiprofen

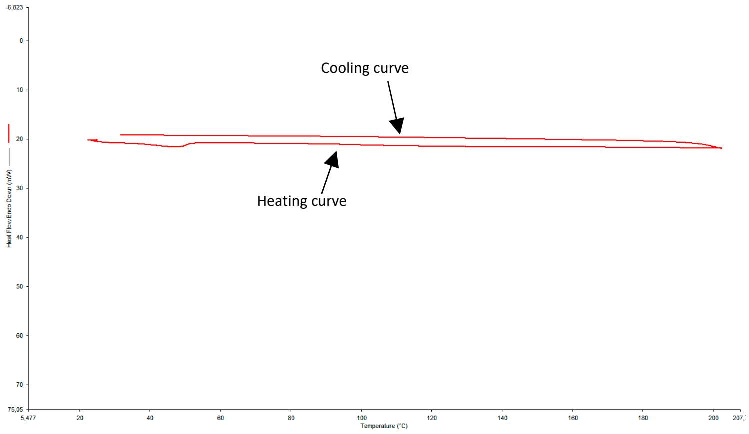

Figure S1.13. extrudate containing Eudragit RL, stearic acid, talcum and 25% m/m flurbiprofen

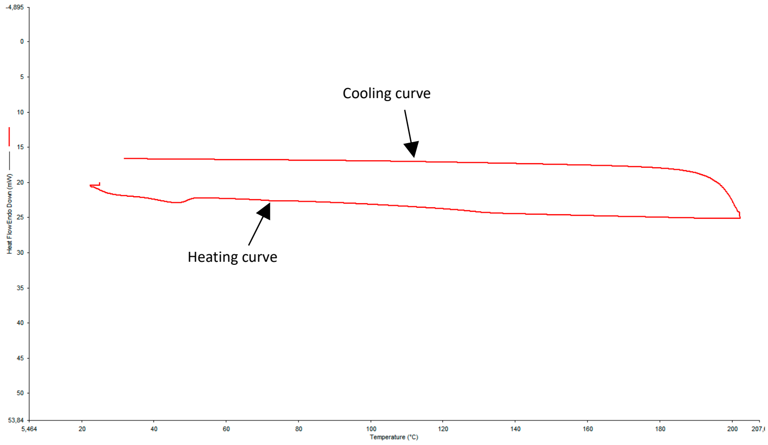

Figure S1.14. extrudate containing Eudragit RL, stearic acid, talcum and 30% m/m flurbiprofen

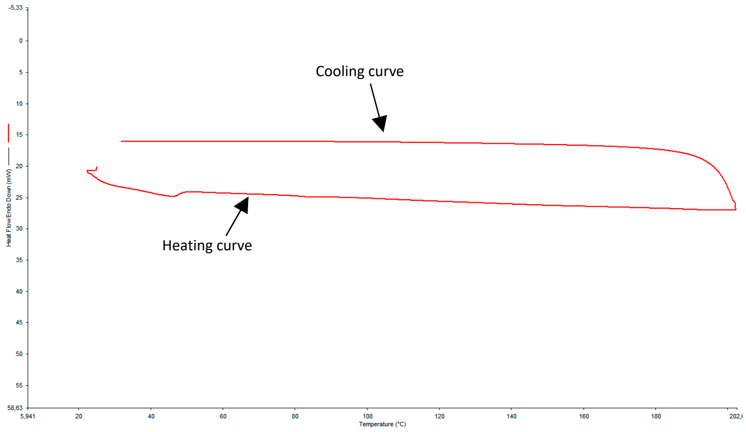

Figure S1.15. extrudate containing Eudragit RL, stearic acid, talcum and 35% m/m flurbiprofen
